# Evidence for Right-Sided Horses Being More Optimistic than Left-Sided Horses

**DOI:** 10.3390/ani8120219

**Published:** 2018-11-22

**Authors:** Isabell Marr, Kate Farmer, Konstanze Krüger

**Affiliations:** 1 Department Equine Economics, Faculty Agriculture, Economics and Management, Nuertingen-Geislingen University, Neckarsteige 6-10, 72622 Nuertingen, Germany; konstanze.Krueger@hfwu.de; 2 Behavioural Physiology of Farm Animals, University of Hohenheim, Garbenstr. 17, 70599 Stuttgart, Germany; 3 School of Psychology & Neuroscience, University of St Andrews, St Mary’s Quad, South Street, St Andrews, Fife, Scotland KY16 9JP, UK; katefarmer@utanet.at; 4 Zoology/Evolutionary Biology, University of Regensburg, Universitaetsstraße 31, 93053 Regensburg, Germany

**Keywords:** cognitive bias, motor, sensory, laterality, optimism, pessimism, judgment task, horse

## Abstract

**Simple Summary:**

Behaviour that indicates stress or poor welfare in horses can be very subtle and, especially for the lay person, difficult to assess. Furthermore, the absence of such behaviour does not necessarily indicate a healthy mental state. Therefore, this study aimed to investigate whether a preference for the left or right forelimb in different tasks (motor laterality) or for left or right sensory organs (sensory laterality) indicates a positive mental state (positive cognitive bias—optimism) or negative mental state (negative cognitive bias—pessimism). This study demonstrates that horses that use the right forelimb more often when starting to move off from a standing position (initial forelimb use) are more likely to expect a neutral stimulus to be positive and to be in an optimistic mental state than horses that use the left forelimb. This knowledge about the horses’ mental state can help us to improve their welfare by minimizing negative events. Furthermore, evaluation of the mental state of animals by determining motor laterality is quicker and easier than conventional tests for cognitive bias that include a long period of training.

**Abstract:**

An individual’s positive or negative perspective when judging an ambiguous stimulus (cognitive bias) can be helpful when assessing animal welfare. Emotionality, as expressed in approach or withdrawal behaviour, is linked to brain asymmetry. The predisposition to process information in the left or right brain hemisphere is displayed in motor laterality. The quality of the information being processed is indicated by the sensory laterality. Consequently, it would be quicker and more repeatable to use motor or sensory laterality to evaluate cognitive bias than to perform the conventional judgment bias test. Therefore, the relationship between cognitive bias and motor or sensory laterality was tested. The horses (*n* = 17) were trained in a discrimination task involving a box that was placed in either a “positive” or “negative” location. To test for cognitive bias, the box was then placed in the middle, between the trained positive and negative location, in an ambiguous location, and the latency to approach the box was evaluated. Results indicated that horses that were more likely to use the right forelimb when moving off from a standing position were more likely to approach the ambiguous box with a shorter latency (generalized linear mixed model, *p* < 0.01), and therefore displayed a positive cognitive bias (optimistic).

## 1. Introduction

Emotions are closely associated with cognition. Cognition can initiate emotions, and emotions influence cognition, resulting in a cognitive bias [1], which leads to an enhanced positive or negative perception or expectation of neutral stimuli. Positive and negative emotions evolved to support the search for valuable resources and to prevent the organism from harm [2,3]. They are the result of attention, perception, and memory [3].

The measurement of cognitive bias can help in the assessment of animal welfare, as the absence of stress parameters does not necessarily indicate a healthy mental state. Unpredictable or unenriched housing has been shown to reduce anticipation of positive events (negative cognitive bias—pessimism) in rats [4,5] and starlings [6], while the change to enriched housing or release from a stressful situation results in enhanced anticipation of positive events (positive cognitive bias—optimism) in non-human primates [7], rats [8], sheep [9], pigs [10], and horses [11,12]. Specific stereotypic behaviours that are indicators of poor animal welfare are associated with a negative cognitive bias in starlings [13] and mice [14]. The measurement of cognitive bias in animals by conditioning them to discriminate between a positive and negative stimulus is time-consuming and limited by the learning effect relating to the ambiguous stimulus, resulting in low repeatability [15]. Therefore, other parameters are needed.

Motor and sensory laterality may be such indicators. They describe the preferred use of the left or right forelimbs and sensory organs, respectively. In common marmosets, the perception of an ambiguous stimulus as positive has been associated with right-handedness and the perception of an ambiguous stimulus as negative or threatening with left-handedness when picking up food in a relaxed situation [16]. This indicates a link between cognitive bias and motor laterality. The limbs and sensory organs (with the exception of the olfactory organs) are connected to the contralateral brain hemisphere [17]. The right brain hemisphere mainly controls withdrawal behaviour and responses to stress, novelty, social interactions, and predators, and is connected to the left side, whereas the left hemisphere is generally responsible for categorization of stimuli, routine situations, and approach behaviour and is connected to the right side [18,19,20,21,22,23]. The observation of motor laterality indicates the predisposition to process information in the left or right brain hemisphere [16]. Therefore, motor laterality may be an indicator for cognitive bias when neutral stimuli are presented. Individuals that prefer the left forelimb may have a more pessimistic outlook and treat an ambiguous stimulus as threatening, and individuals that prefer the right forelimb may have a more optimistic outlook and treat the ambiguous stimulus as positive. Sensory laterality is an indicator for brain specialization of perceptual functions [24] and is more flexible, changes faster, and more situation-related than motor laterality [25]. Therefore, it may be a better predictor for cognitive bias, as it better displays the spontaneous reaction to a novel, ambiguous object and indicates the hemisphere that was used first to process the incoming information, according to the individual’s positive or negative expectations.

Horses (*Equus caballus*) display motor and sensory laterality depending on their breed [20], age [26], social interaction [23], stress load [25], emotionality [27,28], and the type of incoming or processed information [29]. The strength of motor laterality may also depend on conformation characteristics [30]. Both sensory and motor laterality are easy to assess: motor laterality can be observed while the horse is grazing on pasture or eating hay, and sensory laterality can be tested in a novel object test. Furthermore, horses’ laterally positioned eyes and ears facilitate the evaluation of sensory laterality. 

The traditional measurement of cognitive bias is restricted due to a learning effect that has been demonstrated in sheep which show significantly fewer approaches to an ambiguous stimulus in repeated tests [15]. In comparison, the assessment of laterality is not restricted and is less time consuming. Therefore, the aim of the study was to test whether motor and/or sensory laterality is related to cognitive bias. Seventeen horses were trained in a discrimination task to test for cognitive bias using laterality as the parameter. As different methods of measuring motor laterality have been shown not to correlate in their results for preferred forelimb use in horses [22], three different methods were implemented and compared (forelimb position in a relaxed situation, forelimb position in a task-related situation, and the initial forelimb used when starting to move). 

The following questions were addressed: Which measurement of laterality correlates with cognitive bias, and does right-sidedness indicate a positive cognitive bias?

## 2. Materials and Methods

### 2.1. Animals and Location

The study was conducted during winter and spring 2017/2018 with 17 domestic horses at three different facilities (facility 1: N = 5; facility 2: N = 4; facility 3: N = 8) in Nuertingen, Germany. There were ten geldings and seven mares of different types: warmblood (N = 10), pony (N = 6), and thoroughbred (N = 1). As motor laterality starts to increase at two years of age [26], subjects aged three to 26 years (with a median of 13 years) were tested. We assumed the subjects would include a broad range of left-sided, right-sided, and ambilateral horses. The horses were housed either in group housing (N = 8, five horses at facility 1 and three at facility 3), or in individual boxes with access to paddocks at any time, pasture, and contact with conspecifics (N = 9, four horses at facility 2 and five at facility 3). The horses’ individual needs for roughage and supplementary feed were covered. All horses were in healthy condition (no lameness, no acute illness) and had been familiar with general handling from the ground for at least one year. They were either unridden, trained only from the ground, or ridden for a maximum of one hour per day. 

### 2.2. Experimental Procedure

The training and testing of cognitive bias was adapted from previous cognitive bias studies on horses [11,12]. The horses were habituated to the experimental box and learned to discriminate two locations: (a) with a reward (“positive location”) and (b) with no reward (“negative location”). Afterwards they were tested for their expectations of a positive event [31] when confronted with an a third location (“ambiguous location”). In the following three days of training and testing, the motor laterality of each horse was observed and the sensory laterality was calculated from a novel object test.

### 2.3. Cognitive Bias Test

#### 2.3.1. Apparatus and Test Arena for Cognitive Bias

A wooden box with a lid was used (30 cm × 30 cm × 22 cm) and magnets held the lid shut. The horses could open the box by pushing the lid with their muzzles, and in the case of the positive location, they would then be able to eat the carrot inside. The lid could be locked by inserting a stick underneath it, which prevented the horse from opening the box and reaching the carrot inside. The box always contained a carrot, but it was set up to be openable at the positive location and to remain locked at the negative and ambiguous locations. The experiment was carried out either in an indoor or outdoor riding arena, in a fenced area of 20 × 20 m. The box was placed 4 m to the left or right of the midline and 10 m from a starting line, so that the horses had to walk 10 m to each location (right, middle, left). The starting line was marked with two poles laid on the ground to the left and right and a space of 1.5 m (start point) between them for the horses to walk through. All trials were recorded with a camera (Panasonic Lumix DMC-TZ8, Panasonic Marketing Europe GmbH, Wiesbaden, Germany) behind a cover, in direct line with the starting point and the ambiguous location.

#### 2.3.2. Habituation, Habituation Area, and Training

The habituation of the horses to the experimental box was conducted separately for each horse either in the individual’s own paddock for the horses living in paddock boxes, or in the grooming area for those in group housing. First, the horse was allowed to eat pieces of carrot from the box with the lid open. Then, in the training phase, a carrot was placed in the box and the lid was closed. The training criterion was reached when the horse opened the box and ate the carrot three times consecutively.

There were 10 training sessions, each consisting of six trials: three with the box in a positive and three in a negative location. The locations were in a pseudorandom order with no more than two consecutive trials with the box in the same location, with the exception of the first training session. In the first session, all horses received the same order (two trials positive location, two trials negative location, one trial positive location, one trial negative location). The horses were randomly assigned to have either the positive location on the left (N = 8) or on the right (N = 9).

At the start of a trial, experimenter 1 led the horse on a halter and lead rope to the starting point, where it was stopped at a 90° angle to the start line with its forelimbs on the starting line. Experimenter 1 then turned her back to the experimental setup, removed the lead rope from the horse’s halter, and stood still. When the horse was free to move it was allowed 60 s to approach the box. If the horse did not approach the positive location, open the box, and eat the carrot within 60 s on the first training day, experimenter 1 led the horse to the box and allowed it to open the box and eat the carrot. If the horse did not approach the negative location within 60 s on the first training day, experimenter 1 led the horse to the box and allowed it to investigate the box for about 5 s. After each trial, experimenter 1 caught the horse, led it back to the starting line, and held it facing away from the experimental set up. Then, experimenter 2 walked to the box and refilled it with a carrot if necessary, and/or relocated it (according the pseudorandom order for each horse), before the next trial started. Experimenter 2 also recorded all trials with the camera.

#### 2.3.3. Test

Before the horses were confronted with the box in the ambiguous location (i.e., in the middle, between the positive and negative location) they were confronted once with the positive and the negative location of the box for repetition, and randomly assigned to either positive (N = 6) or negative (N = 11) location first. To test the cognitive bias, the box was locked and placed in the ambiguous location. When the horses approached the ambiguous location, we observed the latency of the approach and for how long the horses investigated and tried to open the box within the 60 s. To avoid habituation and learning effects [15,32], the horses were tested only once with the ambiguous location and the horses’ spontaneous reactions were recorded.

#### 2.3.4. Analysis of the Horses’ Performance

The analyses were based on the video recordings. To establish whether the horses learned to discriminate between the positive and the negative locations in the training trials, latencies were measured from the time of removal of the lead rope at the starting position until the first contact with the box. If the horse did not approach the box within the 60 s, a latency of 60 s was recorded. The horse was considered to have learned to discriminate between the negative and the positive locations when the latency to approach the positive location was significantly shorter than for the negative location in training sessions 8 to 10. When testing the horses’ approach to the ambiguous location, the latency was measured in the same way as for the training. For horses that approached the ambiguous box within the 60 s, we observed how long they investigated the box (total time the horse was in contact with the box, i.e., nose, muzzle, hoof). Those that approached the box were categorized as optimistic and those who did not approach the box were categorized as pessimistic.

### 2.4. Laterality

#### 2.4.1. Motor Laterality

Motor laterality was assessed independently in three different ways. Firstly, we measured motor laterality in a relaxed situation [21] and termed it “relaxed forelimb position”. We observed how often the left or right forelimb was placed in front while eating hay in the box and/or grazing on pasture in a relaxed situation. A lateral position was documented when one of the front feet was one hoof length or more in front of the other. Otherwise, an ambilateral forelimb position was documented. Sixty observations per horse were spread over three days and conducted at 30 s intervals. The motor laterality was observed once after the last two training sessions and once after the testing session.

A second motor laterality measurement (termed “initial forelimb use”) was taken from the video recordings of the cognitive bias training. We observed which forelimb was used by each horse when it moved off from the starting point [33]. This yielded a maximum of 60 observations and a minimum of 53 for each horse, depending on how many times they moved away from the start point.

A third motor laterality evaluation was measured (termed “task-related forelimb position”), again from video recordings of the cognitive bias training. We observed which forelimb was initially placed in front or whether horses stood ambilaterally while opening or investigating the box. A lateral position was documented when one of the front feet was one hoof length or more in front of the other, otherwise an ambilateral forelimb position was documented [30]. This yielded a maximum of 56 observations and a minimum of 45 observations for each horse, depending on the number of times they ate from or investigated the box.

Laterality indices (LIs) were calculated separately for all three measurements and for each horse [21,34]. The formula LI = (R − L)/(R + L) was used for the initial forelimb use and the formula LI = (R − L)/(R + L + A) was used for relaxed forelimb position and task-related forelimb position. R describes the number of observations of right forelimb use, L the number of observations of left forelimb use, and A the ambilateral use of both forelimbs. A positive LI indicates a preference for the right forelimb and a negative LI indicates a preference for the left forelimb.

#### 2.4.2. Sensory Laterality

Sensory laterality was observed by ad libitum sampling for each horse during a novel object test, conducted either in its paddock or in the grooming area, following the cognitive bias tests. Novel objects were placed individually one to two meters in front of the horse and the side of the head initially used to investigate the object (left, right, or ambilateral) was observed. Lateral sensory organ use was counted when the horse approached the object with a divergence of more than 5° from a straight line (i.e., from a 90° positioning to the object), turned the head more than 5° when inspecting the object, or clearly touched the object with a specific side of the head for inspection with the eye, nose, or ear. The horses were confronted with a total of nine objects in a random order over three days. The objects were a yellow/black (random pattern) cone, a ball, a plastic bottle filled with water, a folded blue plastic tarpaulin, a yellow swimming noodle, a coloured tube, a box with white and red striped barrier tape, a piece of white polystyrene, and a folded silver windscreen cover (all sized 20 cm to 70 cm in height). A laterality index (LI) was calculated as described for the relaxed forelimb position and task-related forelimb position (see Section 2.4.1. Motor laterality) using the following formula: LI = (R − L)/(R + L + A).

### 2.5. Inter-Observer Reliability

Inter-observer reliability was calculated from two observers’ analysis of eight horses’ latency to approach the box (training: Spearman’s rho = 0.95, and testing: Spearman’s rho = 0.98), of the three measurements of motor laterality (when grazing/hay eating (relaxed forelimb position): Spearman’s rho = 0.97, when starting to move off from a standing position (initial forelimb use): Spearman’s rho = 0.86, and at the experimental box (task-related forelimb position): Spearman’s rho = 0.88), and of the sensory laterality observation (Spearman’s rho = 0.97).

### 2.6. Statistical Analysis

RStudio (version 0.99.484, Boston, MA, USA) and the package R commander (version 2.2.1) were used for the statistical analysis. Figures were constructed with Microsoft Excel 2010 and the cs-tool of Microsoft Excel (Microsoft Corporation, Washington, DC, USA). As the data were not normally distributed (Shapiro-Wilk test: most *p* < 0.05), non-parametric tests were used. The Spearman rank correlation was used to test whether the three different measurements of motor laterality were correlated. The Wilcoxon signed rank test for repeated measurement was used to analyse whether the horses learned to discriminate between the positive and the negative locations by comparing the latency to approach the two boxes. A Wilcoxon signed rank test for repeated measurement was used to compare the time spent investigating the ambiguous box with the mean time spent investigating the box at the negative location in the last three training sessions. A generalized linear mixed model (GLMM) with random effects (age, facility, breed, housing, sex) and fixed effects (positive side, last location before testing with ambiguous location) was used to test whether the latency to approach the ambiguous box was affected by the fixed or random factors, i.e., the laterality measurement: formula = latency to approach ambiguous box ~ positive side + last location before testing with ambiguous location + laterality/(age + facility + breed + housing + sex), family = Gaussian (identity). Non-significant random factors were removed step-wise to simplify the models and to improve the models’ goodness of fit only when their deletion did not cause a significant reduction of the models’ goodness of fit. The results are presented after model simplifications with the best goodness of fit. The complete full models and the reduced model are presented in the supplementary materials (Results S1). All tests were two-sided. The significance level was set at 0.05.

## 3. Results

### 3.1. Correlation between the Three Measurements of Motor Laterality

No significant correlations between the three measurements of motor laterality (relaxed forelimb position, initial forelimb use, and task-related forelimb position) were found (Spearman N = 17, all *p* > 0.05). However, there was a weak correlation trend between the relaxed forelimb position and the task-related forelimb position (Spearman N = 17, rho = 0.42, *p* = 0.09). We therefore proceeded to analyse the test parameters separately for each motor laterality measurement.

### 3.2. Training Criterion

All horses reached the training criterion of learning to distinguish between the positive and the negative locations of the feed box; the latency to approach the positive location (median = 8 s) was significantly shorter than the latency to approach the negative location (median = 60 s) in all horses (Wilcoxon test N = 9, V < 3.5, *p* < 0.05 for all horses).

### 3.3. Latency to Approach Ambiguous Box and Laterality

**Figure 1:**
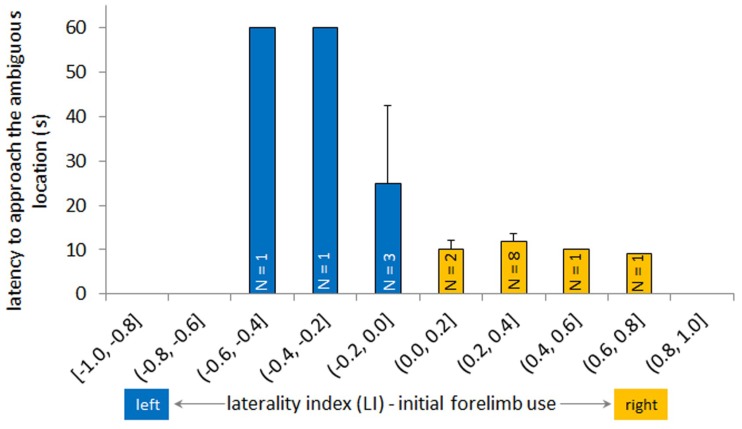
Mean latency to approach the test box in the ambiguous location compared to the initial forelimb use. Horses with a laterality index of the initial forelimb use lower than 0 (blue) started walking from the starting position more often with the left forelimb, and horses with an index higher than 0 (yellow) started more often with the right forelimb (GLMM: ambiguous box ~ initial forelimb use, N = 17, *t* = −3.71, *p* = 0.002). Horses which needed more than 60 s were considered not to have made a spontaneous decision (i.e., three horses: one horse at (−0.6, −0.4], one horse at (−0.4, −0.2], one horse at (−0.2, 0.0]). The mean latency and the standard error are shown. GLMM: generalized linear mixed model.

Horses that were faster to approach the ambiguous box showed a significantly higher laterality index in the initial forelimb use, indicating that they were more right-sided in their forelimb choice when starting to walk (GLMM: ambiguous box ~ initial forelimb use, N = 17, *t* = −3.71, *p* = 0.002, Figure 1). Furthermore, horses in facility 1 (group housing, N = 5) that were quicker to approach the ambiguous box also had a significantly higher sensory laterality index, i.e., they were more lateralized to right sensory organ use (GLMM: ambiguous box ~ sensory laterality/(facility), N_facility_ b = 5, t_facility_ b = −2.73, p_facility_ b = 0.02). 

However, there was no significant relationship between the latency to approach the ambiguous box and the relaxed forelimb position (GLMM: ambiguous box ~ relaxed forelimb position, N = 17, *t* = 0.38, *p* = 0.71) or the task-related forelimb position (GLMM: ambiguous box ~ task-related forelimb position, N = 17, *t* = −0.82, *p* = 0.42), nor was there any relationship across all facilities between the latency to approach the ambiguous box and the horses’ sensory laterality (GLMM: ambiguous box ~ sensory laterality/(facility), N = 17, *t* = 1.00, *p* = 0.34).

### 3.4. Time Spent Investigating the Ambiguous Box

**Figure 2:**
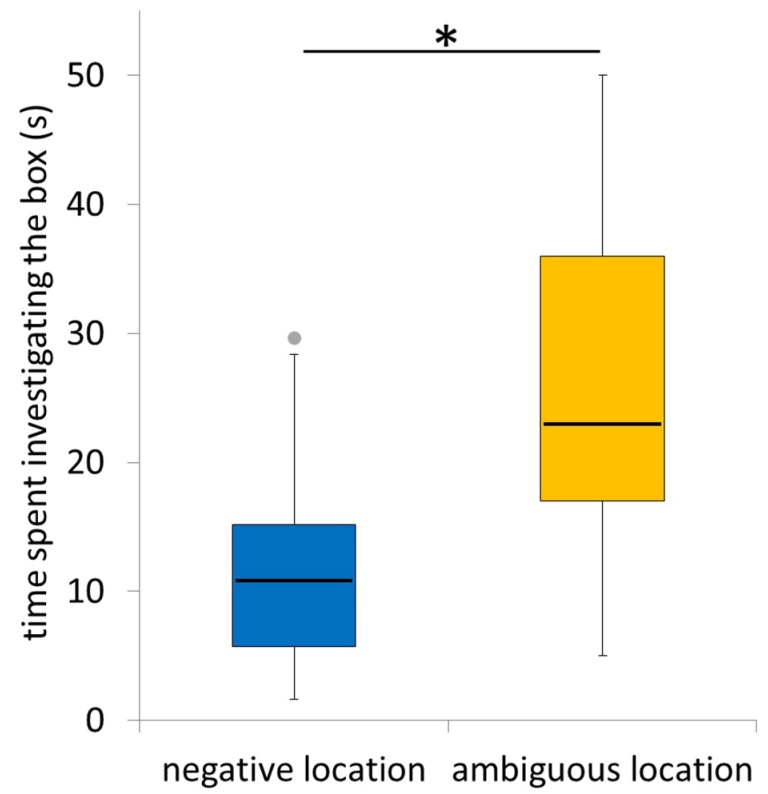
Time spent investigating the box at the ambiguous location (i.e., the novel location where the horses did not know whether it could be opened) compared with mean time spent investigating the box at the negative location (i.e., the location where the horses knew it was locked) during the last three training sessions (N = 14). * *p* < 0.05.

Fourteen horses approached the ambiguous box, and the time spent investigating it was significantly longer than the time spent investigating the negative box during the last three training sessions (Wilcoxon test N = 14, V = 92, *p* = 0.01, Figure 2).

## 4. Discussion

Horses that used the right forelimb more often when starting to move from the starting position (initial forelimb use) were more likely to treat the ambiguous box as positive and to approach it. The horses that approached the box at the ambiguous location seemed to expect an unlocked box that would allow them to eat the carrot inside. They investigated and tried to open the box for significantly longer than when the box was at the negative location, demonstrating an optimistic manner. Therefore, these horses can be considered more optimistic than horses that used the left forelimb more often. The latter hesitated or did not approach the box within the given time of 60 s. In common marmosets, the handedness in a relaxed situation (picking up food) is correlated with a cognitive bias [16]. In horses, cognitive bias was not related to the relaxed forelimb position or to the task-related forelimb position; it therefore remains debatable whether motor laterality measurement can be compared between species [35]. In future research on horses, a careful choice should be made on the method used to assess motor laterality, as the three methods of the present study did not correlate, as already demonstrated in other studies [22]. The forelimb preference seems to be dependent on the task, situation, and the strength of laterality on conformation characteristics [30].

Sensory laterality may not be a reliable measure of an overall cognitive bias in horses, as it changes too quickly and is too flexible [25]. The effect of the facility on the cognitive bias and the sensory laterality may have been a result of management, environment, and/or environmental changes, and these factors require further investigation. Nonetheless, further research is needed to answer the question of whether right-sided sensory laterality predicts a more optimistic manner in horses in general, or whether the strength of sensory laterality predicts the emotionality of a horse, as a preference for left sensory organs has been observed in both positive and negative situations [23,28,29].

Cognitive bias testing using a judgment bias test is limited because of the learning effect [15] and the time-consuming training procedure; as such, the measurement of initial forelimb use may be a promising method for assessing cognitive bias in horses, and especially in the repeated assessment of cognitive bias. Furthermore, the use of a judgment bias test to test for cognitive bias has limited application in the assessment of animal welfare because the cognitive bias training/testing procedure is a release from the investigated stressful situation. That stress release may result in a positive cognitive bias, even though the stress hormones may still be elevated from the investigated stressful situation (sheep: [9], horses: [11]). It remains to be seen whether a stress-induced left-shift in motor laterality [25] is indicative of the development of a pessimistic cognitive bias, and which type of motor laterality measurement may be a more reliable parameter. In rats, there is a link between vulnerability to stress-induced pessimism and cognitive bias [36]. Therefore, motor laterality may be a promising indicator not only of the horses’ cognitive bias but also of the vulnerability to stress-induced pessimism, and may be helpful in the selective breeding of less stress-prone horses and in improving animal welfare. This needs to be investigated in future research. Another interesting topic for future research would be whether the initial forelimb choice can be manipulated, e.g., by positioning the forelimbs in special relation to each other (standing square, left or right forelimb advanced), as in the present study we did not manipulate or train the horses to stop and stand in a special manner.

## 5. Conclusions

A preference for the use of the right forelimb when moving off from a standing position (initial forelimb use) indicates an optimistic manner/positive cognitive bias in horses in specific context and object investigation. There was no relation between the cognitive bias and the other laterality measurements (task-related forelimb position, relaxed forelimb position, sensory laterality). The knowledge of the animals’ cognitive bias, and therefore its emotions, can help to improve welfare by enabling negative events to be minimized [2], but further research is needed on the accurate measurement of motor laterality.

## Supplementary Materials

The following are available online at http://www.mdpi.com/2076-2615/8/12/219/s1, Results S1: Generalized linear mixed model with random effects (complete and simplified models).
