# Supplementary material for: Evidence for Right-Sided Horses Being More Optimistic than Left-Sided Horses
Source: Animals (Basel). 2018 Nov 22;8(12):219. doi: 10.3390/ani8120219 (PMC6315450; doi:10.3390/ani8120219)
Supplement: Supplementary file 1 [file animals-08-00219-s001.pdf]

Generalized linear mixed model with random effects

```
LatAmb:       latency to approach ambiguous location in s
Facility:     facility a, b, and c
BoxbeforeAmb:last location before testing with ambiguous location; positive (p) or
             negative (n)
Breed:        warmblood, pony, and thouroughbred
Housing:      group housing or box with paddock
Pos.Side:     trained positive location of the box; left (l) or right (r)
Sex:          mare (m) or gelding (g)
ML:           motor laterality (laterality index)
SL:           sensory laterality (laterality index)
```

Initial forelimb use (MLstart):

```
glm(formula = LatAmb ~ PosSide + BoxBeforeAmb + MLstart/(Age +
    Facility + Breed + housing + Sex), family = gaussian(identity),
    data = Datenmatrix)

Deviance Residuals:
     Min        1Q    Median        3Q       Max
-19.7084   -6.1635    0.9422    4.4886   25.9218

Coefficients:
                                 Estimate Std. Error t value Pr(>|t|)
(Intercept)                        19.519     12.199   1.600    0.161
PosSide[T.r]                       10.539     18.094   0.582    0.581
BoxBeforeAmb[T.p]                  14.282     13.821   1.033    0.341
MLstart                          -156.998    121.395  -1.293    0.243
MLstart:Age                         5.541      4.778   1.160    0.290
MLstart:Facility[T.f1]            -87.303     96.268  -0.907    0.399
MLstart:Facility[T.f2]            -24.721     88.628  -0.279    0.790
MLstart:Breed[T.Thouroughbred]     60.591    107.757   0.562    0.594
MLstart:Breed[T.Warmblood]          6.856     68.645   0.100    0.924
MLstart:housing[T.group housing]  100.889    111.959   0.901    0.402
MLstart:Sex[T.m]                   13.059     40.735   0.321    0.759

(Dispersion parameter for gaussian family taken to be 339.5188)

    Null deviance: 6291.5  on 16  degrees of freedom
Residual deviance: 2037.1  on  6  degrees of freedom
  (61 observations deleted due to missingness)
AIC: 153.61
Number of Fisher Scoring iterations: 2
```

Simplified model with the best goodness of fit

```
Call:
glm(formula = LatAmb ~ MLstart, family = gaussian(identity),
    data = Datenmatrix)

Deviance Residuals:
     Min        1Q    Median        3Q       Max
-24.4113   -8.2052   -0.5927    8.9316   27.2017
```

```
Coefficients:
            Estimate Std. Error t value Pr(>|t|)
(Intercept)   27.089      4.155   6.519  9.7e-06 ***
MLstart      -44.858     12.083  -3.713  0.00208 **
---
Signif. codes:  0 '***' 0.001 '**' 0.01 '*' 0.05 '.' 0.1 ' ' 1

(Dispersion parameter for gaussian family taken to be 218.5852)

    Null deviance: 6291.5  on 16  degrees of freedom
Residual deviance: 3278.8  on 15  degrees of freedom
  (61 observations deleted due to missingness)
AIC: 143.7

Number of Fisher Scoring iterations: 2
```

Relaxed forelimb position (MLrelaxed):

```
Call:
glm(formula = LatAmb ~ PosSide + BoxBeforeAmb + MLrelaxed/(Age +
    Breed + Facility + housing + Sex), family = gaussian(identity),
    data = Datenmatrix)

Deviance Residuals:
     Min        1Q    Median        3Q       Max
-23.8939  -11.8452    0.1796    5.7358   30.3379

Coefficients:
                                   Estimate Std. Error t value Pr(>|t|)
(Intercept)                          18.737     13.411   1.397    0.212
PosSide[T.r]                         11.832     17.116   0.691    0.515
BoxBeforeAmb[T.p]                   -17.632     17.974  -0.981    0.364
MLrelaxed                            21.675     88.385   0.245    0.814
MLrelaxed:Age                        -4.991      4.500  -1.109    0.310
MLrelaxed:Breed[T.Thouroughbred]    -72.389    106.153  -0.682    0.521
MLrelaxed:Breed[T.Warmblood]         28.317     55.439   0.511    0.628
MLrelaxed:Facility[T.f1]             80.599    143.591   0.561    0.595
MLrelaxed:Facility[T.f2]            121.926     78.234   1.558    0.170
MLrelaxed:housing[T.group housing]  -28.689    148.233  -0.194    0.853
MLrelaxed:Sex[T.m]                  -28.335     58.245  -0.486    0.644

(Dispersion parameter for gaussian family taken to be 604.7097)

    Null deviance: 6291.5  on 16  degrees of freedom
Residual deviance: 3628.3  on  6  degrees of freedom
  (61 observations deleted due to missingness)
AIC: 163.42

Number of Fisher Scoring iterations: 2
```

Simplified model with the best goodness of fit

```
Call:
glm(formula = LatAmb ~ MLrelaxed, family = gaussian(identity),
    data = Datenmatrix)
```

```
Deviance Residuals:
    Min       1Q   Median       3Q      Max
-15.280  -10.191   -9.438   -1.028   42.896

Coefficients:
            Estimate Std. Error t value Pr(>|t|)
(Intercept)    18.27       5.63   3.246  0.00543 **
MLrelaxed       5.01      13.21   0.379  0.70978
---
Signif. codes:  0 '***' 0.001 '**' 0.01 '*' 0.05 '.' 0.1 ' ' 1

(Dispersion parameter for gaussian family taken to be 415.4507)

    Null deviance: 6291.5  on 16  degrees of freedom
Residual deviance: 6231.8  on 15  degrees of freedom
  (61 observations deleted due to missingness)
AIC: 154.62

Number of Fisher Scoring iterations: 2
```

Task related forelimb position (MLbox):

```
Call:
glm(formula = LatAmb ~ PosSide + BoxBeforeAmb + MLbox/(Sex +
    housing + Facility + Breed + Age), family = gaussian(identity),
    data = Datenmatrix)

Deviance Residuals:
     Min        1Q    Median        3Q       Max
-16.9130   -5.7045    0.2229    4.9464   27.4710

Coefficients:
                                   Estimate Std. Error t value Pr(>|t|)
(Intercept)                          25.913      9.571   2.707   0.0352 *
PosSide[T.r]                          3.113     10.250   0.304   0.7716
BoxBeforeAmb[T.p]                   -22.437     13.700  -1.638   0.1526
MLbox                              -229.231    140.779  -1.628   0.1546
MLbox:Sex[T.m]                      408.932    171.137   2.389   0.0541
MLbox:housing[T.group housing] -313.324    207.044  -1.513   0.1810
MLbox:Facility[T.f1]                257.044    196.064   1.311   0.2378
MLbox:Facility[T.f2]                288.342    188.371   1.531   0.1767
MLbox:Breed[T.Thouroughbred]       -468.070    229.614  -2.039   0.0876
MLbox:Breed[T.Warmblood]            180.220    139.136   1.295   0.2428
MLbox:Age                             1.513      7.744   0.195   0.8516
---
Signif. codes:  0 '***' 0.001 '**' 0.01 '*' 0.05 '.' 0.1 ' ' 1

(Dispersion parameter for gaussian family taken to be 322.9118)

    Null deviance: 6291.5  on 16  degrees of freedom
Residual deviance: 1937.5  on  6  degrees of freedom
  (61 observations deleted due to missingness)
AIC: 152.75
Number of Fisher Scoring iterations: 2
```

Simplified model with the best goodness of fit

```
Call:
glm(formula = LatAmb ~ MLbox, family = gaussian(identity), data = Datenmatrix)

Deviance Residuals:
    Min       1Q   Median       3Q      Max
-17.561  -11.128   -7.712   -0.362   40.232

Coefficients:
            Estimate Std. Error t value Pr(>|t|)
(Intercept)   21.128      5.349    3.95  0.00128 **
MLbox        -15.295     18.644   -0.82  0.42486
---
Signif. codes:  0 '***' 0.001 '**' 0.01 '*' 0.05 '.' 0.1 ' ' 1

(Dispersion parameter for gaussian family taken to be 401.4253)

    Null deviance: 6291.5  on 16  degrees of freedom
Residual deviance: 6021.4  on 15  degrees of freedom
  (61 observations deleted due to missingness)
AIC: 154.03

Number of Fisher Scoring iterations: 2
```

Sensory laterality

```
Call:
glm(formula = LatAmb ~ BoxBeforeAmb + PosSide + SL/(Sex + housing +
    Facility + Breed + Age), family = gaussian(identity), data = Datenmatrix)

Deviance Residuals:
    Min       1Q   Median       3Q      Max
-20.891   -2.226    0.958    5.185   18.954

Coefficients:
                            Estimate Std. Error t value Pr(>|t|)
(Intercept)                   18.512      8.051   2.299   0.0611
BoxBeforeAmb[T.p]            -16.591     12.878  -1.288   0.2451
PosSide[T.r]                   3.316     11.099   0.299   0.7752
SL                            49.811     98.999   0.503   0.6328
SL:Sex[T.m]                 -112.856     66.097  -1.707   0.1386
SL:housing[T.group housing]   72.000    115.301   0.624   0.5553
SL:Facility[T.f1]           -155.273    100.962  -1.538   0.1750
SL:Facility[T.f2]            -78.376     44.225  -1.772   0.1267
SL:Breed[T.Thouroughbred]    137.191    141.769   0.968   0.3706
SL:Breed[T.Warmblood]        -64.578     74.220  -0.870   0.4177
SL:Age                         2.529      3.187   0.794   0.4577
---
Signif. codes:  0 '***' 0.001 '**' 0.01 '*' 0.05 '.' 0.1 ' ' 1

(Dispersion parameter for gaussian family taken to be 224.4657)

    Null deviance: 6291.5  on 16  degrees of freedom
Residual deviance: 1346.8  on  6  degrees of freedom
  (61 observations deleted due to missingness)
```

```
AIC: 146.57

Number of Fisher Scoring iterations: 2
```

Simplified model with the best goodness of fit

```
Call:
glm(formula = LatAmb ~ SL/(Facility), family = gaussian(identity),
    data = Datenmatrix)

Deviance Residuals:
    Min       1Q   Median       3Q      Max
-23.639   -7.606   -4.575    6.328   33.378

Coefficients:
                 Estimate Std. Error t value Pr(>|t|)
(Intercept)         17.61       4.33   4.066  0.00134 **
SL                  15.09      15.08   1.001  0.33508
SL:Facility[T.f1]  -96.24      35.29  -2.727  0.01728 *
SL:Facility[T.f2]  -53.92      27.60  -1.954  0.07256
---
Signif. codes:  0 '***' 0.001 '**' 0.01 '*' 0.05 '.' 0.1 ' ' 1

(Dispersion parameter for gaussian family taken to be 267.899)

    Null deviance: 6291.5  on 16  degrees of freedom
Residual deviance: 3482.7  on 13  degrees of freedom
  (61 observations deleted due to missingness)
AIC: 148.72

Number of Fisher Scoring iterations: 2
```
